# Intravitreal triamcinolone for management of idiopathic juxtafoveolar telangiectasis

**DOI:** 10.4103/0974-620X.60023

**Published:** 2010

**Authors:** Nitin Nema, Michael S. Ip

**Affiliations:** Department of Ophthalmology, Sri Aurobindo Institute of Medical Sciences, Indore, India,; 1 University of Wisconsin, Madison, WI, USA

Idiopathic juxtafoveolar retinal telangiectasis (IJRT) group 1A usually affects one eye of young and middle aged male patients.[1,2] Telangiectatic and aneurysmal retinal vessels are typically located temporal to the foveola. Visual loss is associated with macular edema or hard exudates extending into the foveal center from the abnormal leaky retinal capillaries.[1] Laser treatment to the telangiectatic capillaries may be effective in reducing foveolar edema and improving vision.[1–3] We report the case of a patient with unilateral IJRT group 1A that was refractory to focal laser photocoagulation treatment but was managed successfully with intravitreal triamcinolone acetonide injection.

**Figure 1:**
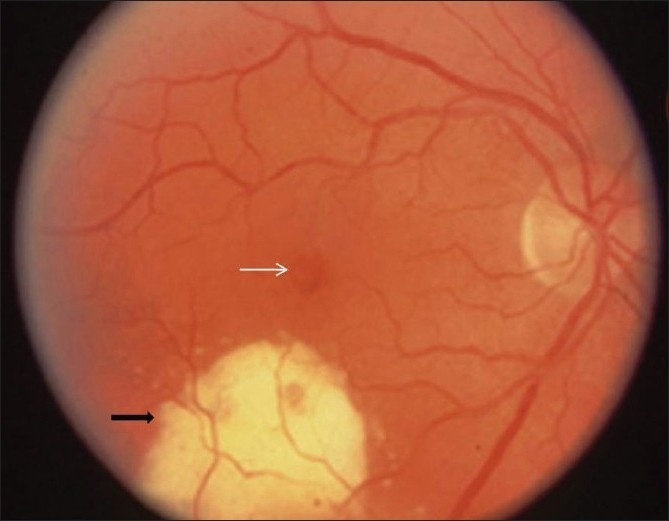
Fundus photograph of OD shows telangiectatic blood vessels and hard exudates temporal and inferior to fovea (black arrow). Cystic foveal changes are also noted (white arrow)

**Figure 2:**
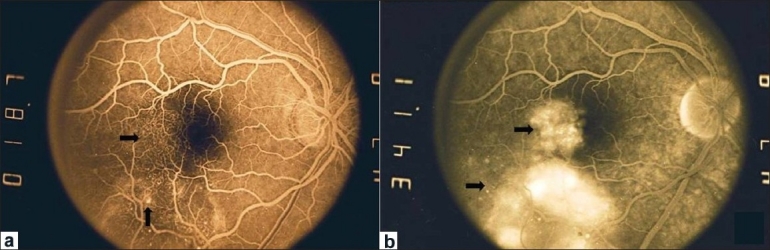
Fluorescein angiography of OD shows (a) Juxtafoveolar and inferotemporal telangiectatic capillaries (arrow) in early phase and (b) Leakage of dye (arrow) corresponding to areas of telangiectasia in late phase

A 40-year-old healthy male was referred to us for visual distortion and inability to see in the upper left field of vision in the right eye (OD) for six weeks. Medical work-up and laboratory tests excluded the presence of any systemic ailment including hypertension and diabetes mellitus. The best-corrected visual acuity was 20/30 OD and 20/20 in the left eye (OS). Anterior segment examination showed no cell or flare in the anterior chamber or in the anterior vitreous in both eyes (OU). Intraocular pressure (IOP) was 15 mmHg OU. Dilated fundus examination OD showed abnormal telangiectatic blood vessels and hard exudates temporal to the fovea as well as cystic appearance in the foveal area [Figure 1]. The fundus examination OS was unremarkable. Fluorescein angiography (FA) revealed dilated telangiectatic retinal capillaries in the temporal retina in the early phase of the angiogram and leakage of dye in the central and temporal portions of the foveal avascular zone in the late phases [Figure 2]. Thickened temporal retina and intraretinal cystic spaces within the fovea were seen on optical coherence tomography (OCT) of the right eye. The central foveal thickness measured on OCT was 279 micrometers. The fundus picture on slit-lamp biomicroscopic ophthalmoscopy, indirect ophthalmoscopy and digital colored photography, and findings of FA and OCT were consistent with the diagnosis of IJRT group 1A. Laser photocoagulation was done, focally ablating the leaky telangiectatic capillaries.

**Figure 3:**
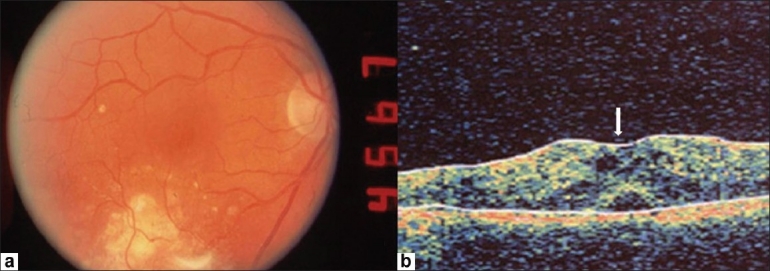
(a) Fundus photograph of OD shows an increase in macular edema 15-month following laser photocoagulation. (b) Corresponding optical coherence tomography image shows loss of normal foveal contour and inner retinal foveal cysts (arrow)

**Figure 4:**
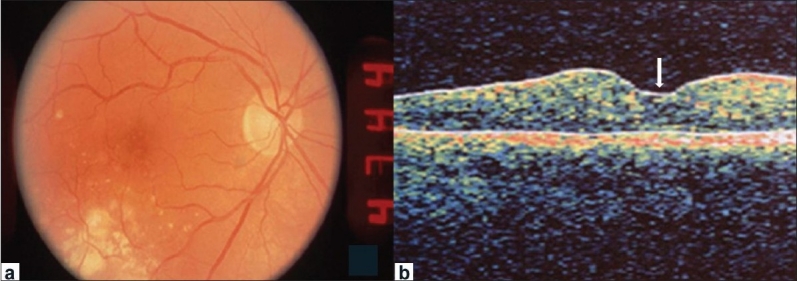
(a) Fundus photograph OD two weeks following intravitreal injection of triamcinolone shows significant improvement in macular edema. (b) Corresponding optical coherence tomography shows reduction in foveal cystic spaces and restoration of a nearly normal foveal architecture (arrow)

Over the next one year the patient was followed closely without improvement in vision (following laser treatment) or change in the fundus appearance. However, 15 months after the initial examination, his vision dropped to 20/50 OD with significant increase in macular edema clinically and on OCT [Figure 3]. The central foveal thickness measured was 433 micrometers. In view of the prior poor response to laser photocoagulation, the patient was counseled for a trial of intravitreal injection of steroid. An informed written consent was obtained from the patient. An intravitreous injection of 4 mg/0.1 cc of triamcinolone acetonide (Kenalog, Bristol-Myers-Squibb, Princeton, NJ) was given via the pars plana 4 mm posterior to the limbus in the inferotemporal quadrant OD. The eye remained quiet with no slit-lamp signs of intraocular inflammation during the immediate post-injection period. Follow up, two weeks later, showed improvement in visual acuity to 20/30 with significant improvement in macular edema OD both on clinical examination and OCT [Figure 4]. The central foveal thickness reduced to 281 micrometers. This improvement was maintained at the one year follow-up evaluation post-treatment. IOP transiently got elevated to 28 mmHg OD but was successfully controlled in six weeks with topical timolol maleate.

On the basis of biomicroscopic and fluorescein angiographic patterns, Gass and Blodi have classified IJRT into three groups:[1] Group 1 patients have unilateral easily visible retinal telangiectasis and exudation, that is further divided into subgroup 1A with congenital temporal telangiectasis and subgroup 1B with idiopathic focal telangiectasis of early onset. Group 2 patients have bilateral telangiectasia with visual loss due to macular edema or subretinal neovascularization. Patients in this group often show right angle venules, superficial retinal crystalline deposits and plaques of retinal pigment hyperplasia. Group 3 patients have easily visible telangiectasis and parafoveal capillary closure (group 3A) or may be associated with central nervous system vasculopathy (group 3B).

Laser photocoagulation and photodynamic therapy of the telangiectatic capillaries in IJRT group 1A are reported to be effective in reducing the foveolar edema and exudation.[2–4] However, in our patient, the laser treatment was ineffective in controlling the progression of disease and visual deterioration.

There was dramatic improvement in vision two weeks following intravitreal triamcinolone injection in the present case. The vision improved along with resolution of macular edema that was confirmed on clinical examination and on OCT. Li *et* al. and Maia *et* al. successfully treated their patients of group 1 IJRT with intravitreal injection of triamcinolone acetonide.[5,6] Both the patients in the two reports showed improvement in the visual acuity, and decrease in macular edema and retinal thickness on OCT. Intravitreal steroids may be helpful due to their anti-inflammatory effects and stabilization of the inner blood-retinal barrier through reduction in vascular permeability.

Our experience with this patient demonstrates that intravitreal triamcinolone acetonide may be considered an additional therapeutic option besides photocoagulation in the management of IJRT group 1A, especially when the latter treatment is ineffective. Moreover, OCT is a useful non-invasive tool that helps in diagnosing the disease and following it over the years. Further long-term case controlled prospective studies evaluating the safety and efficacy of this treatment modality for patients with IJRT group 1A are warranted.
